# The zebrafish *lysozyme C *promoter drives myeloid-specific expression in transgenic fish

**DOI:** 10.1186/1471-213X-7-42

**Published:** 2007-05-04

**Authors:** Chris Hall, Maria Vega Flores, Thilo Storm, Kathy Crosier, Phil Crosier

**Affiliations:** 1 Department of Molecular Medicine and Pathology, School of Medical Sciences, The University of Auckland, Auckland, New Zealand

## Abstract

**Background:**

How different immune cell compartments contribute to a successful immune response is central to fully understanding the mechanisms behind normal processes such as tissue repair and the pathology of inflammatory diseases. However, the ability to observe and characterize such interactions, in real-time, within a living vertebrate has proved elusive. Recently, the zebrafish has been exploited to model aspects of human disease and to study specific immune cell compartments using fluorescent reporter transgenic lines. A number of blood-specific lines have provided a means to exploit the exquisite optical clarity that this vertebrate system offers and provide a level of insight into dynamic inflammatory processes previously unavailable.

**Results:**

We used regulatory regions of the zebrafish *lysozyme C *(*lysC*) gene to drive enhanced green fluorescent protein (EGFP) and DsRED2 expression in a manner that completely recapitulated the endogenous expression profile of *lysC*. Labeled cells were shown by co-expression studies and FACS analysis to represent a subset of macrophages and likely also granulocytes. Functional assays within transgenic larvae proved that these marked cells possess hallmark traits of myelomonocytic cells, including the ability to migrate to inflammatory sources and phagocytose bacteria.

**Conclusion:**

These reporter lines will have utility in dissecting the genetic determinants of commitment to the myeloid lineage and in further defining how lysozyme-expressing cells participate during inflammation.

## Background

A major challenge faced by researchers in various fields of immunology is understanding the cellular interactions that govern a successful immunological response. A typical vertebrate immune response depends upon the highly orchestrated migration and motility of various hematopoietic compartments and their subsequent interactions that ultimately control the magnitude of the response [1,2]. Current techniques used to study the cellular dynamics of such processes include intravital microscopy and tissue explant assays [3,4]. Although extremely informative, both methods rely upon invasive surgical procedures and do not represent a true whole animal setting. The zebrafish is emerging as an extremely promising model system to study specific aspects of disease mechanisms [5-11]. Not only is the early zebrafish embryo optically clear, it is also highly amenable to genetic manipulation and small chemical screens [11,12]. These attributes have been exploited to study aspects of immune system development [13-16].

Zebrafish, like mammals experience two separate waves of hematopoiesis. The first 'primitive wave' generates embryonic erythrocytes as well as early myeloid-derived macrophages and neutrophils from two distinct intraembryonic compartments, the caudally-located intermediate cell mass (ICM) and a rostral blood-forming region derived from cephalic mesoderm [17-19]. Whether these early myeloid cell populations are specifically embryonic or persist to contribute to more adult immune cell populations is unclear. The second, 'definitive' wave, believed to initiate within the ventral wall of the dorsal aorta eventually gives rise to all adult blood lineages, including erythrocytes, macrophages, several types of granulocytes, lymphocytes and thrombocytes [18,20,21]. Zebrafish also possess a B and T cell repertoire as part of a functional adaptive immune system; a *rag*-dependent system that emerged with the evolution of the jawed vertebrates [14,22].

Macrophages represent the main differentiated cell of the monocyte phagocyte system and an essential primary innate barrier to infection [23,24]. Their primary function is phagocytosis and they are widely distributed throughout the organism, displaying variations in behavior and morphology [23]. Despite these variations, most macrophages can be defined by the expression of certain enzymes, such as lysozyme and peroxidase [23]. In addition to phagocytosis of infectious materials, macrophages also function during host tissue repair, regulation of inflammatory events (through secretion of cytokines), regulation of immune responses (through antigen presentation to lymphocytes) and are believed to influence certain aspects of neovascularization [23,25,26].

In zebrafish, embryonic macrophage precursors originate from the rostral blood compartment and derive from lateral plate mesoderm [27]. During early somitogenesis, anterior lateral plate mesoderm converges to the midline and eventually lies just beneath the paraxial mesoderm. From this region, macrophage precursors expressing the early myeloid marker *pu.1 *migrate to initially populate the anterior yolk surface and then more posterior yolk regions [17,27-31]. The transcription factor *pu.1 *has been demonstrated, by whole mount in situ hybridization, to mark the myeloid lineage up to 30 hours post fertilization (hpf) [17,32]. Once on the yolk, these primitive macrophages begin to express genes encoding the actin-binding protein L-plastin and lysozyme C [17,28,29,33]. Upon initiation of embryonic circulation, a subset of macrophages enter the circulation and are distributed throughout the embryonic tissues. This is in contrast to neutrophils, where appearance on the yolk is preceded by that within the posterior ICM, as revealed by expression of the *myeloperoxidase *(*mpo*) [17,19], suggesting a degree of anatomical separation during embryonic monocytopoiesis and granulopoiesis.

To generate a macrophage reporter line, and facilitate direct observation of macrophage events in situ in real-time, we have generated novel transgenic lines in which the zebrafish *lysC *promoter was used to drive macrophage expression of EGFP and DsRED2. Lysozyme is a cationic antibacterial enzyme capable of hydrolyzing specific linkages within the bacterial cell wall [34]. In humans, lysozyme is synthesized within both granulocytic and monocytic cells [34,35]. Regulatory regions of the mouse *lysozyme M *gene, one of two *lysozyme *genes found in mice [36], have been demonstrated to be sufficient to drive myelomonocytic-specific expression of the EGFP fluorescent reporter [37,38]. In zebrafish, *lysC *expression has been reported to specifically mark the macrophage compartment, based upon initial expression within cells on the yolk and co-localised expression with *L-plastin *[33]. We used the highly efficient *Tol2 *transposon system to generate germline *lysC::EGFP *and *lysC::DsRED2 *transgenic founders [39]. Approximately 6.35 kb of promoter sequence was sufficient to drive reporter expression in a manner that recapitulated the endogenous expression profile of *lysC *and was consistent with the known ontogeny of the macrophage lineage. Inflammation, bacterial infection and phagocytic assays demonstrated that these labeled cells possessed hallmark traits of the macrophage lineage while time-lapse confocal microscopy revealed their highly dynamic morphology, proliferative potential and ability to roll along vascular endothelia. Within transgenic embryos, labeled cells expressed markers of the macrophage lineage, including *L-plastin*. Some fluorescent cells also contained transcripts for myeloperoxidase (*mpo*), raising questions regarding the macrophage-specificity of *lysC *previously reported [33], but consistent with the presence of lysozyme within both macrophages and granulocytes of mammals. In summary, we present the *lysC::EGFP *and *lysC::DsRED2 *reporter lines as having marked myelomonocytic compartments that will prove useful in defining the determinants of lineage commitment during myelopoiesis and in examining the contribution of innate immune cells during inflammatory events.

## Results and Discussion

### Strategy to generate a zebrafish *lysozyme C *reporter line

The *lysC *promoter region was selected for generation of a reporter line, as expression of the *lysC *gene had been described as being specific to the zebrafish macrophage lineage [33]. To facilitate comparison with the *lysC *transgenic reporter lines generated in this work, we have included a detailed expression analysis of *lysC *during embryonic/larval development that extends previous studies (see Additional file 1: *lysC *expression during early embryonic/larval development). A zebrafish genomic BAC clone was initially identified in silico as containing sequence of the Ensembl-predicted *lysC *gene. This BAC clone (I.D. zC250A24) was confirmed via amplification and sequence verification as containing the first coding exon of *lysC*. Fragments corresponding to sequence upstream of the initiation codon were cloned from this BAC (for details see Methods section) into EGFP- and DsRED2-encoding pT2KXIGΔin *Tol2*-containing vectors [39]. This generated the pT2K/lysC::EGFP and pT2K/lysC::DsRED2 expression vectors. To verify that these constructs could drive expression of the fluorescent reporters to simulate endogenous *lysC*, we injected 1-cell stage zebrafish embryos with 30 pg of either construct (injection of higher doses resulted in toxicity). EGFP and DsRED2 expression was first observed exclusively on the yolk surface with EGFP first detectable around 24 hpf and DsRED2 approximately 4 hours later. This is most likely due to the longer time required for proper folding of the DsRED2 tetrameric protein (data not shown). A subset of these cells entered the circulation and could be seen moving over the yolk ventrally towards the embryonic heart. This expression pattern is consistent with the endogenous expression pattern of *lysC*, as detected by whole mount in situ hybridization as shown here and by others [33] and the ontogeny of early macrophage development [27].

These constructs were then injected into 1-cell stage embryos along with capped *transposase *transcript to induce transposition and generate germline transgenic founders. Six germline transgenic founders were identified for both the *lysC::EGFP *and *lysC::DsRED2 *lines. Both lines displayed correct Mendelian inheritance ratios in subsequent generations and identical reporter expression profiles of comparable intensities (data not shown).

Next we describe, in detail, reporter expression within stable *lysC::EGFP *transgenic animals. It is of note that the *lysC::DsRED2 *line possessed an identical expression profile throughout development (see Additional file 2: Co-localized expression of EGFP and DsRED2 within *lysC::EGFP*/*lysC::DsRED2 *compound transgenic larvae), albeit commencing slightly later.

### EGFP expression within *lysC::EGFP *transgenic embryos recapitulates the endogenous expression profile of *lysC *that traces the early development of the macrophage lineage

**Figure 1:**
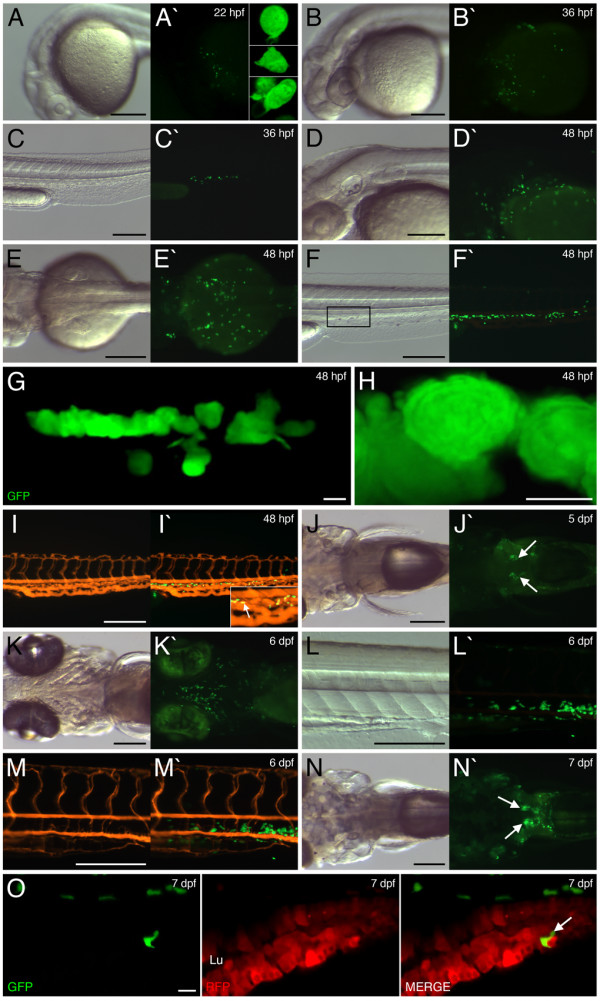
**EGFP expression within *lysC::EGFP *transgenic embryos and larvae**. EGFP expression within 22 hpf (A), 36 hpf (B and C), 48 hpf (D-I), 5 dpf (J), 6 dpf (K-M) and 7 dpf (N and O) transgenic embryos/larvae. (A, B and D) Lateral views of developing head, anterior to left. (C, F-I, L and M) Lateral views of trunk/tail region, anterior to left. (E, J and N) Dorsal view of cranio-trunk region, anterior to left. (K) Ventral view of head region, anterior to left. (A-N and A'-N') Bright field and dark field views, respectively. Insets in A' and I' represent magnified views of A' and I', respectively. (G) Summed Z stacks through aggregates of EGFP-labeled cells within posterior ICM region (marked by box in F). (H) Magnified view of cells in G. (I and M) Microangiography using red fluorescent microspheres within 48 hpf and 6 dpf transgenic larvae, respectively. (I' and M') Images merged with EGFP expression. Arrow in I' denotes EGFP-labeled cell within caudal vascular plexus. Arrows in J' and N' denote expression within the pronephric glomerulus. (O) Summed Z stacks through mid-intestine of 7 dpf *lysC::EGFP/I-FABP::RFP *compound transgenic larva (anterior to left). Abbreviations: Lu, gut lumen. Scale bars: 200 μm in A-F and I-N; 10 μm in G and O; 5 μm in H.

EGFP expression within the *lysC::EGFP *line was first detectable by fluorescence microscopy at 22 hpf. This expression was restricted to a small collection of weakly expressing cells located over the anterior yolk (Fig. 1A). This location is consistent with the onset of *lysC *expression described here (see Additional file 1: *lysC *expression during early embryonic/larval development) and by others [33]. This location also corresponds with lineage tracing of primitive macrophages using DIC optics [27]. Summed Z stacks using confocal microscopy through individual EGFP-positive cells within 22 hpf transgenic embryos revealed that these early putative macrophages could be divided into three main classes. One comprised cells possessing a rounded morphology with very few, if any, filopodia (Fig. 1A'; upper inset). The second class possessed a more compacted morphology with ruffled borders (Fig. 1A'; middle inset). Cells in the third class were more elongated and possessed long filopodia (Fig. 1A'; lower inset). These morphologically distinct classes are consistent with previous observations made through DIC imaging of similarly staged live zebrafish embryos and likely represent stages of macrophage differentiation and/or activation [27]. This initial domain of putative macrophage development preceded the onset of embryonic circulation. Upon initiation of circulation at approximately 25 hpf, a subset of EGFP-positive cells together with primitive erythrocytes was noted to migrate ventrally through the duct of Cuvier to the embryonic heart. In comparison to erythrocytes, these labeled cells demonstrated an ability to resist blood flow, often pausing for a certain period before resuming movement. This suggests that these cells interact with the overlying epidermis and/or the underling yolk syncytial layer. This ability to interact with these surfaces and undertake environmental sensing has been observed using Nomarski microscopy [27]. The primitive circulatory network then transported these labeled cells to the posterior of the embryo where they began to populate the posterior ICM compartment. By 36 hpf, larger numbers of EGFP-expressing cells were seen over the yolk (with this domain expanded to also include more posterior yolk regions), a subset of which migrated with erythrocytes (Fig. 1B). Also at 36 hpf, EGFP-positive cells were seen accumulating within the caudal vasculature and surrounding mesenchyme (Fig. 1C). EGFP expression progressively intensified and by 48 hpf large numbers of strongly expressing cells were observed within the head mesenchyme and over the yolk (Fig. 1D and 1E). This developmental stage also marked a massive expansion of the posterior expression domain with large numbers of EGFP-expressing cells clustered together in close vicinity to the caudal vascular plexus (Fig. 1F). Summed Z stacks through EGFP-labeled cells within this domain demonstrated that they clustered in tight aggregates and in general did not display features of activated macrophages such as dynamic cell morphology and extended filopodia (Fig. 1G and 1H). Time-lapse confocal microscopy confirmed this region to be an active proliferative domain for this marked cell population (see Additional file 3: Movie of dividing EGFP-labeled cell within ICM compartment of 52 hpf *lysC::EGFP *larva). Microangiography using red fluorescent microspheres revealed that EGFP-positive cells within the ventral tail region were largely excluded from the caudal vascular plexus and occupied mesenchymal tissue between the vascular endothelia (Fig. 1I). This observation supports a recent study in which this mesenchymal ventral tail domain has been termed the caudal hematopoietic tissue and represents a newly defined region of hematopoietic proliferative activity [21]. However, a small subset of cells were located within the tail blood vessels, and these cells were observed to navigate the caudal vascular plexus, seemingly to crawl along the endothelial walls (Fig. 1I'; inset). To image this rolling in greater detail we performed time-lapse confocal microscopy within *lysC::DsRED2*/*fli1::EGFP *double transgenic larvae. DsRED2-expressing cells were clearly seen rolling along the interior endothelial surface of the vasculature (see Additional file 4: Movie of labeled cell rolling along vasculature endothelia). From 5 dpf, a new domain of EGFP-expression commenced in the pronephric glomerulus (Fig. 1J), consistent with *lysC *expression (see Additional file 1: *lysC *expression during early embryonic/larval development). The first evidence of hematopoietic activity in the zebrafish kidney, the equivalent of mammalian bone marrow, occurs around 4 dpf where hematopoietic progenitors are closely associated with renal vasculature [18,40]. Within 6 dpf transgenic larvae, EGFP-positive cells were observed in close association with the pharyngeal skeleton (Fig. 1K) and clusters of EGFP-expressing cells remained in the ventral trunk/tail region (Fig. 1L). Microangiography revealed that these cells were still largely excluded from the trunk vasculature (Fig. 1M). By 7 dpf, EGFP expression within cells marking the glomerulus had significantly expanded (Fig. 1N). When visualized within 7 dpf *I-FABP::RFP *larvae [41], a line where the red fluorescent protein (RFP) is targeted to intestinal epithelia that express intestinal fatty acid binding protein (I-FABP), EGFP-expressing cells were detected migrating between RFP-labeled gut epithelia within the developing intestinal wall (Fig. 1O). This evidence of early mucosal innate immune surveillance is of particular interest given that the gut exists as an externally accessible tube and is subject to microbial influence from as early as 4 dpf [42]. Of note, no ectopic EGFP expression was observed outside of the endogenous expression profile for *lysC*.

In addition to the domains described above, EGFP-labeled cells were observed within the developing brain and retina (see Additional file 5: Labeled cells are located within the developing brain and retina). However, no colonization of the optic tectum by EGFP-expressing cells was seen within the *lysC::EGFP *line throughout embryonic/larval development. Previous studies have demonstrated specific colonization of the optic tectum by *L-plastin*-expressing macrophages [43]. Analysis of our *lysC::EGFP *reporter line and the expression of *lysC *(see Additional file 1, *lysC *expression during early embryonic/larval development) suggest that these cells that mark the optic tectum represent a subset of macrophages that are lysozyme C-deficient. It is of note that within *lysC::EGFP *animals, fewer labeled cells were detected within the developing head when compared with those revealed by *L-plastin *and *fms *expression [43].

### EGFP-expressing cells within *lysC::EGFP *adult fish mark the head kidney and display myelomonocytic morphology

**Figure 2:**
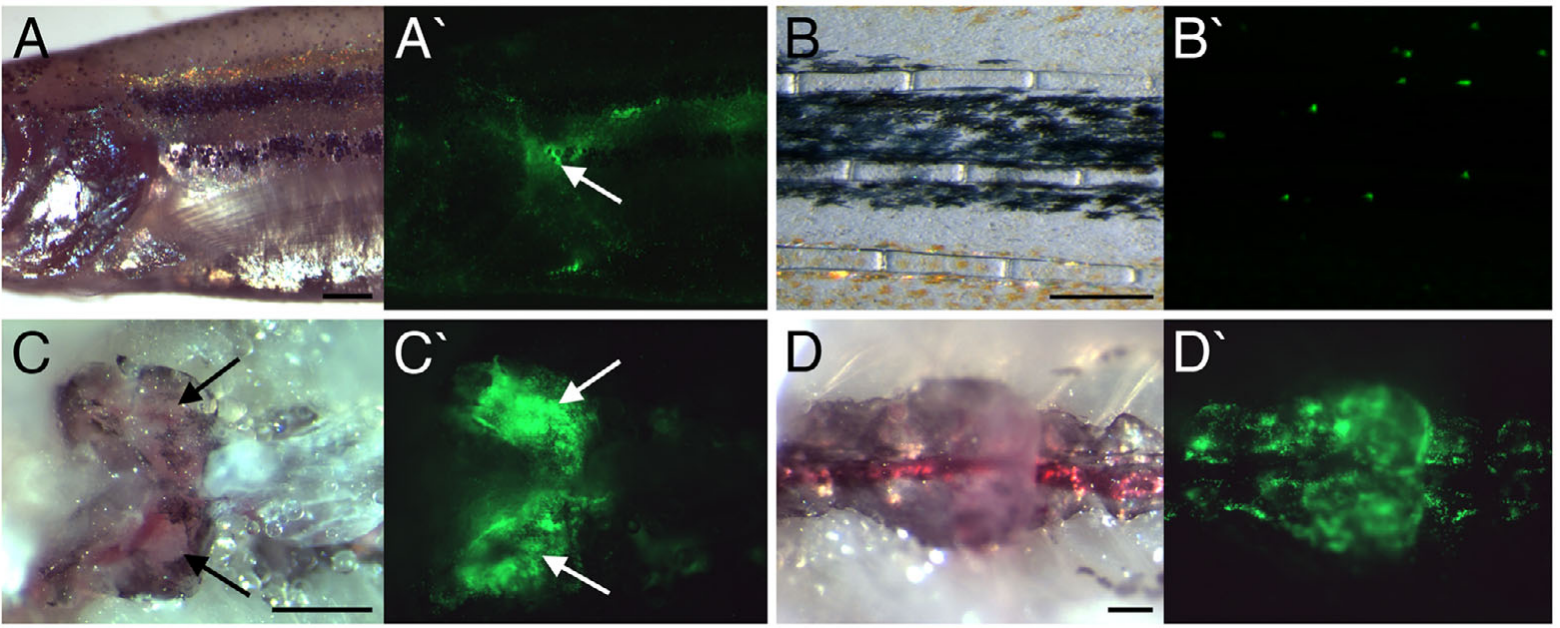
**EGFP expression within adult *lysC::EGFP *transgenic animals**. (A) Lateral view of pectoral fin region. (B) Lateral view of tail fin. (C) Ventral view of dissected head kidney. (D) Ventral view of dissected posterior kidney. Anterior to left in all views. (A-D and A'-D') Bright field and dark field views, respectively. Arrows mark EGFP-expressing cells within the lobes of the head kidney. Scale bars: 500 μm in A, C and D; 250 μm in B.

**Figure 3:**
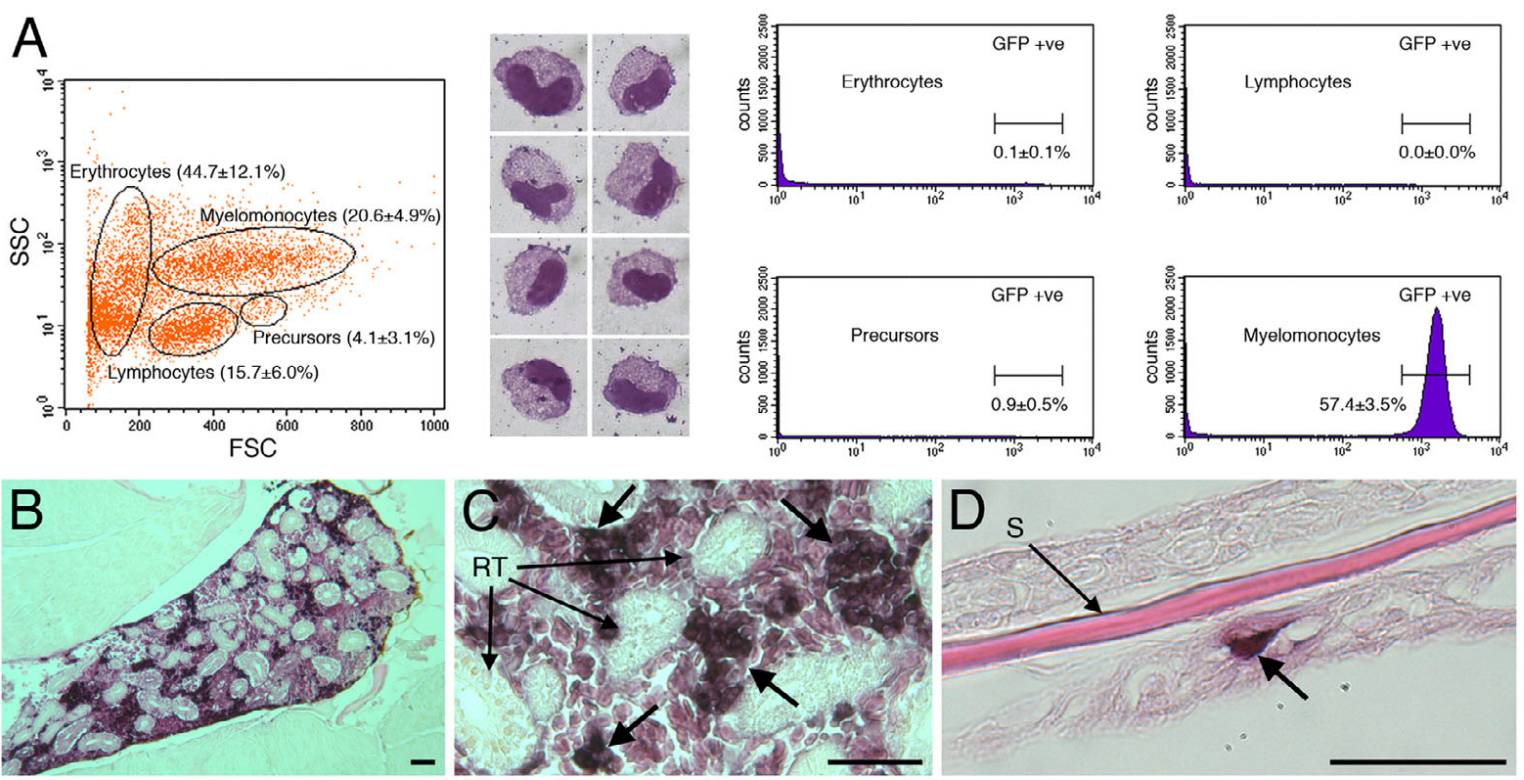
**EGFP-expressing cells within adult kidney are myelomonocytic**. (A) Flow cytometry of definitive hematopoietic compartment from adult *lysC::EGFP *animals by light-scatter characteristics. Percentages represent the relative contribution of the gated compartment. Cytospin preparations of GFP-sorted cells following Leishman's staining show clear myelomonocytic morphology. Analysis of GFP detection demonstrates that the labeled cell population is almost exclusive to the myelomonocytic compartment where 57.4 ± 3.5% (*n *= 4) of cells were GFP-positive. (B-D) Immunohistochemical detection of EGFP within adult *lysC::EGFP *sections. (B and C) Low and high magnification views, respectively, of EGFP-expressing cells (marked by large arrows) located within the head kidney occupying space between the renal tubules. (D) EGFP-expressing cell within the epidermal surface (marked by large arrow). Abbreviations: RT, renal tubule; S, scale. Scale bars: 50 μm in B-D.

When adult transgenic animals were analyzed by fluorescence microscopy, large numbers of EGFP-expressing cells were observed migrating within the epidermal surface of the fish and fins (Fig. 2A and 2B) while others could be seen trafficking through the peripheral vasculature (data not shown). Transgenic adults also displayed intense EGFP expression just posterior to the gills, which appeared to be continuous with expression running in a posterior direction just above the intestine (Fig. 2A). Dissection to expose the kidney revealed this intense fluorescence was emitted by cells located within the head kidney and adjoining posterior kidney (Fig. 2C and 2D). To define the cell compartment expressing EGFP within the kidney we performed FACS analysis and sorting of dissected adult *lysC::EGFP *kidneys. EGFP-labeled cells were found almost exclusively within the myelomonocytic fraction (Fig. 3A), as defined by forward and side scatter characteristics [44]. Approximately 57.4 ± 3.5% (mean ± standard error; *n *= 4) of cells within the myelomonocytic (FSC^hi^SSC^hi^) fraction were marked with EGFP and displayed typical myelomonocytic morphology (Fig. 3A). Previous FACS and morphological analyses have shown monocyte/macrophage cells to constitute approximately 24% of the myelomonocytic fraction of adult zebrafish kidney [44]. This suggests that granulocytic cells, in addition to monocytes/macrophages, are labeled within adult kidneys of *lysC::EGFP *animals. Examination of transverse sections from adult *lysC::EGFP *animals following immunohistochemical detection of EGFP revealed that these labeled cells occupied space between the renal tubules of the kidney (Fig. 3B and 3C), a well characterized definitive hematopoietic site [40]. EGFP-expressing cells were also clearly visible within the fish epidermis (Fig. 3D).

### EGFP-expressing cells are of the myeloid lineage

To confirm that these EGFP-expressing cells within the *lysC::EGFP *line were derived from hematopoietic progenitors, and more specifically were of myeloid descent, transgenic embryos were depleted of Scl and Gata1 by morpholino injection. Depletion of Scl, a transcription factor absolutely required in mammals for both primitive and definitive hematopoiesis [45-47], resulted in an almost complete ablation of EGFP-expressing cells (see Additional file 6: Labeled cells are restricted to the myeloid lineage). This was in contrast to Gata1-depleted embryos which demonstrated marked expansion of the EGFP-marked compartment (see Additional file 6: Labeled cells are restricted to the myeloid lineage) consistent with previous studies in which Gata1-deficient embryos possess an expanded myeloid and diminished erythroid compartment [48].

**Figure 4:**
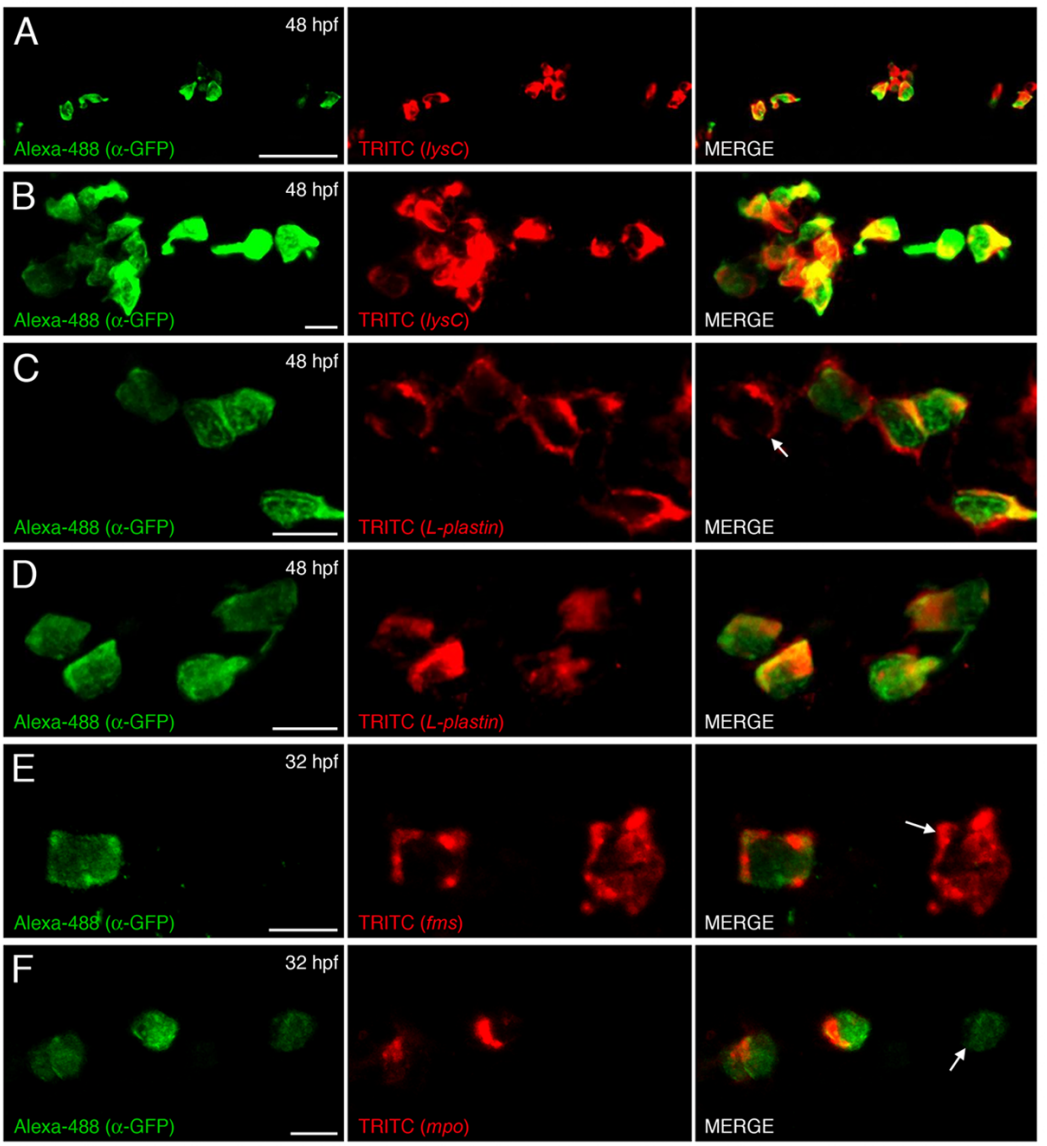
**EGFP-labeled cells co-express myeloid lineage markers**. Dual immunohistological detection of EGFP and in situ hybridization detection of *lysC *(A and B), *L-plastin *(C and D) within 48 hpf and *fms *(E), *mpo *(F) within 32 hpf *lysC::EGFP *animals. (A/C/E/F and B/D) Summed Z stacks within posterior ICM compartment and within hindbrain, respectively, of transgenic animals. Arrow in C and E denotes EGFP-negative cell expressing *L-plastin *and *fms*, respectively. Arrow in F denotes EGFP-expressing cell that does not co-express *mpo*. Anterior to left in all images. Scale bars: 50 μm in A; 10 μm in B-F.

Unlike erythropoiesis, which generates a single mature cell type, myelopoiesis gives rise to monocyte/macrophage cells and several different types of granulocytes. In the zebrafish, a number of markers have been used to distinguish embryonic macrophages, including *L-plastin, draculin *[27], *fms *[43] and *lysC *[33], while neutrophilic granulocytes are characterized by expression of *mpo *[17,19,49,50]. To confirm that EGFP expression was restricted to cells expressing *lysC*, and indirectly that the lysozyme C promoter fragments used to generate the *lysC *reporter lines contained the necessary enhancer elements to faithfully reproduce endogenous *lysC *expression, we performed immunohistological detection of EGFP and whole mount in situ hybridization detection of *lysC*. These experiments confirmed that throughout the entire embryo, all EGFP-expressing cells analyzed possessed *lysC *transcript (Fig. 4A and 4B). A similar result was observed when detecting EGFP and the macrophage marker *L-plastin *[27], in that all EGFP-labeled cells analyzed also expressed *L-plastin *(Fig. 4C and 4D). However, some *L-plastin*-expressing cells were observed that were EGFP negative within the tail (Fig. 4C) as well as the trunk and head regions (data not shown) suggesting that not all macrophages express *lysC*. A subset of EGFP-marked cells were also detected containing *fms *transcript (Fig. 4E). In zebrafish, in addition to marking neural crest precursors of xanthophores, expression of *fms *(which encodes the macrophage colony-stimulating factor receptor) has been demonstrated to co-localize with that of the pan-myeloid marker *pu.1 *within cells considered to be macrophage progenitors on the yolk sac [51]. Expression of *fms *is also observed in more mature macrophage populations [43]. Some labeled cells that expressed *mpo *were detected (Fig. 4F), raising the possibility of neutrophilic identity. Although this is embryonic expression, it is consistent with the FACS analysis previously described where the relative contribution of EGFP-labeled cells within the adult kidney myelomonocytic compartment (57.4 ± 3.5%, *n *= 4) exceeded that previously reported for monocytes/macrophages (24%) [44]. In mammals the expression patterns of lysozyme and myeloperoxidase are dynamic within the hematopoietic compartment [34,35,52-54]. In general, the monocyte/macrophage lineage appears to first synthesize lysozyme followed by myeloperoxidase while the reverse is observed in differentiating neutrophils [35]. In zebrafish, studies have shown specific expression of *mpo *only within morphologically identified granulocytic neutrophils and not within the macrophage lineage [19] leading to the notion that, in zebrafish, *mpo *may represent a specific marker of neutrophils [50]. Furthermore, the initial site of *mpo *expression, in the posterior ICM compartment [17,19], is distinct from that of the macrophage markers *lysC *and *L-plastin *that first appear over the yolk surface [17,33]. However, prior to the onset of circulation, *mpo*-expressing cells are also located over the yolk surface suggesting a separate anterior domain of granulopoiesis [17]. Whether these cells represent a subset of primitive macrophages possessing myeloperoxidase activity is unclear. However, in a transgenic *mpo::EGFP *reporter line, a distinct subset of cells expressing EGFP, in low levels, were identified and have been suggested as being macrophages based upon morphology [49]. Furthermore, co-localized expression of *mpo *and the macrophage marker *L-plastin *has been observed in a small subset of myeloid cells within the early zebrafish embryo [17]. Bennett and colleagues reported that the frequency of these co-expressing cells diminishes during later development suggesting a transient existence.

Recently a medaka transgenic line has been described in which a fugu *mpo *promoter fragment is reported to drive macrophage-specific expression [55] illustrating an interesting difference with zebrafish where the same gene is believed to be largely neutrophil-specific [19]. It seems that within the embryonic myeloid compartment of zebrafish, myeloid-restricted genes are subject to dynamic temporal regulation. The expression profile of the labeled cells within *lysC::EGFP *animals described here, in concert with their initial appearance on the yolk surface suggests that populations of macrophages and also some neutrophils are fluorescently marked.

### Labeled cells within transgenic larvae demonstrate a robust response to inflammation and bacterial infection

**Figure 5:**
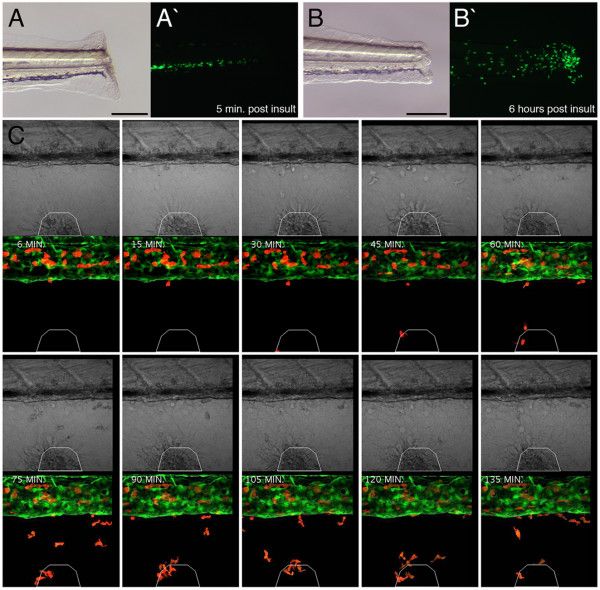
**Labeled cells within transgenic larvae exhibit robust responses to acute inflammation**. (A and B) Inflammation assay within 7 dpf *lysC::EGFP *larva imaged 5 min. and 6 hours post insult, respectively. Lateral views of transected tails, anterior to left. (A/B and A'/B') Bright field and dark field images, respectively. (C) Higher resolution analysis of inflammatory response within 6 dpf *lysC::DsRED2*/*fli1::EGFP *compound transgenic animals following wounding of the ventral fin. Time-lapse images every 15 minutes (starting 6 minutes following wounding) demonstrating progressive accumulation of marked cells at the injury site. White polygon demarcates injury boundaries. Scale bars: 200 μm in A and B.

Embryonic macrophages fulfill a number of roles including phagocytosing cell corpses and protecting the early embryo from bacterial infections [23,27,56-59]. Macrophages are also highly mobile, possessing an ability to migrate through tissues to an inflammatory source [60]. A recent study in zebrafish has shown that an intact microtubule network is a prerequisite for normal macrophage chemotaxis towards a laser-induced injury on the yolk [61]. To address the ability of the labeled cells to migrate in response to acute inflammation, the tails of 7 dpf *lysC::EGFP *larvae were transected and the response of EGFP-expressing cells monitored. As early as 2 hpi (hours post insult), EGFP-labeled cells had accumulated at the injury site and by 6 hpi a significant number of cells had assembled at the trauma region (Fig. 5B). These migratory kinetics are similar to those demonstrated previously for zebrafish leukocyte populations in response to inflammatory insults [19,49,50,61].

**Figure 6:**
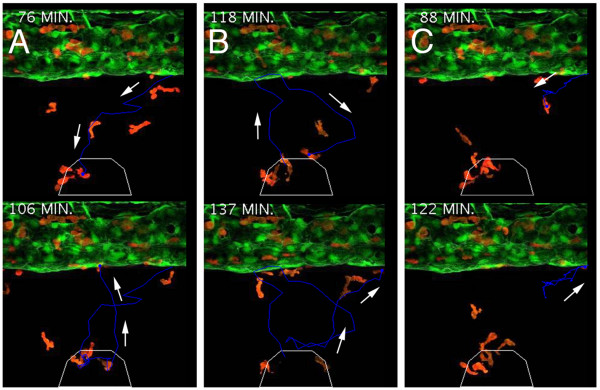
***lysozyme C*-expressing cells demonstrate varied responses to induced inflammation**. (A-C) Cell tracking of individual responses, upper image in A and C displays cells at ventral-most location during migration, lower images display return migration of the same cell towards the caudal vein, arrows denote trajectory. Upper image in B displays a cell revisiting the injury and then retuning to the vasculature (lower image). White polygon demarcates injury boundaries.

To more closely characterize the wound-homing ability of marked cells, we performed time-lapse confocal microscopy within 6 dpf *lysC::DsRED2*/*fli1::EGFP *compound transgenic larvae. A small incision within the ventral fin was sufficient to generate a strong infiltration of DsRED2-expressing cells to the wound (Fig. 5C). In general, migrating cells displayed rapid chemotaxis towards the injury after they had exited the caudal vein. Once at the wound, labeled cells continued migrating both around and within the injured tissue. This analysis revealed three predominant migratory phenotypes. Some cells rapidly migrated to the wound, then after navigating within and around the injury migrated back into the vasculature (Fig. 6A, also see Additional file 7: Movie of tracked cell demonstrating retrograde chemotaxis). Other labeled cells, following their return to the caudal vein were observed exiting once again to revisit to the wound for a second time and subsequently return to the circulation (Fig. 6B, also see Additional file 8: Movie of tracked cell that infiltrates the injury site and returns to the vasculature twice). The third phenotype comprised cells that initially migrated towards the wound only to cease movement for a period of time before returning to the vasculature (Fig. 6C, also see Additional file 9: Movie of tracked cell that fails to infiltrate the wound). In a similar study, marked neutrophils within *mpo::GFP *transgenic zebrafish larvae have been shown to demonstrate a similar retrograde chemotactic response to an induced wound, also within the ventral fin region [49]. This retrograde chemotaxis to the vasculature has been described as a potential mechanism that contributes to the resolution phase of inflammation. Interestingly, neutrophils were observed to migrate almost immediately towards the wound followed by another cell type described morphologically as macrophages [49]. This result supports a macrophage identity for the inflammation-responsive cells in our assay where labeled cells were not observed at the wound until at least 30 minutes following wounding. These studies also support existing knowledge regarding the timing of arrival of the different leukocytic lineages during a robust inflammatory response [60].

**Figure 7:**
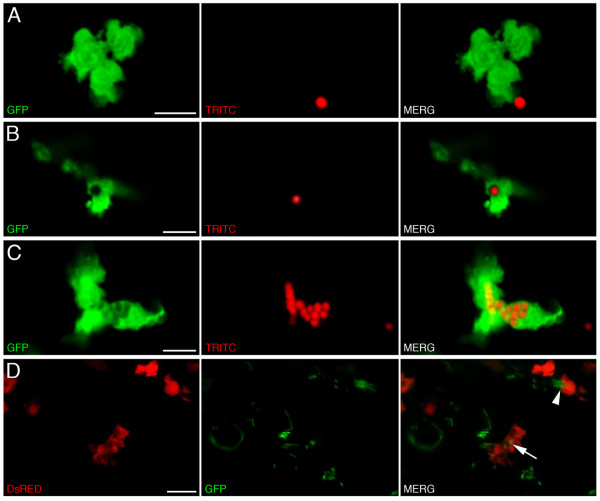
**Labeled cells within transgenic larvae display phagocytic activity**. Phagocytosis of red fluorescent microspheres by labeled cells within 4 dpf *lysC::EGFP *larvae. (A-C) Summed Z stacks through individual EGFP-labeled cells within 4 dpf transgenic larvae approximately 6 hours following injection of fluorescent microspheres. (D) Summed Z stacks through posterior intestine of DsRED2-expressing cells containing ingested GFP-labeled *Salmonella *following infection of *lysC::DsRED2 *larvae at 5 dpf (images captured at 8 dpf). Arrow denotes labeled cell containing GFP-labeled bacteria, arrowhead denotes a labeled cell actively phagocytosing a microcolony of GFP-expressing bacteria. Scale bars: 10 μm.

In addition to expressing a large repertoire of cytokines during infection and wound repair, macrophages are phagocytes that function to clear wounds of cellular debris [60]. To demonstrate that these EGFP/DsRED2-expressing cells were capable of phagocytosis we injected red fluorescent microspheres into 4 dpf *lysC::EGFP *larvae and monitored the uptake of particles by confocal microscopy. Phagocytosis has been defined as the uptake of particles greater than 0.5 μm in diameter [56]. To ensure that phagocytosis was the mechanism of uptake, we selected 2 μm-sized microspheres. Injection of these fluorescent particles, typically just posterior to the swim bladder, resulted in rapid mobilization of EGFP-labeled cells to the site of injection. As early as 6 hours post injection, EGFP-labeled cells were observed in close association with individual microspheres (Fig. 7A). Red fluorescent microspheres were also observed within intracellular phagosomes (Fig. 7B and 7C), confirming their phagocytic potential. Following the injection of fluorescent microspheres and the resulting inflammation, EGFP-labeled cells were observed to be highly active and often physically interacted with each other following the ingestion of fluorescent particles (see Additional file 10: Movie of interacting EGFP-labeled cells within 4 dpf *lysC::EGFP *larva). Using DIC optics in live *Mycobacterium marinum *infection assays, infected macrophages have been observed to transfer phagocytosed bacteria via membrane tethers [57].

During bacterial infections macrophages function to clear invading pathogens and also act as key modulators of both the innate and adaptive immune systems [2]. In zebrafish, macrophages exhibit a robust response to infection [27,57,59]. To examine the response of the marked cell population within the *lysC *reporter lines, we infected *lysC::DsRED2 *zebrafish larvae with GFP-expressing *Salmonella enterica *serovar Typhimurium. Following infection, a robust response was detected within the posterior extremities of the intestine (see Additional file 11: Infection with GFP-expressing *Salmonella *results in a robust inflammatory response within the posterior intestine). Labeled cells within this region were detected containing fluorescent bacteria (Fig. 7D) confirming their contribution to the bacterial inflammatory response.

## Conclusion

Together, these results demonstrate that the *lysC::EGFP*/*DsRED2 *reporter lines mark populations of macrophages and likely also neutrophils. These lines should prove valuable in further dissecting the genetic determinants of lineage commitment during myelopoiesis. They will also have utility in studying the relative contributions to innate immune responses of the leukocyte compartments.

## Methods

### Zebrafish maintenance

Zebrafish (*Danio rerio*) embryos were obtained from natural spawning between *lysC::EGFP*/*DsRED2*, *fli1::EGFP *[62], *I-FABP::RFP *[41] and wild type (obtained from the Zebrafish International Resource Center) adult fish. Embryos were raised at 28°C in Embryo Medium (E3) [63] and developmentally staged as described [64]. Research was conducted with approval from The University of Auckland Animal Ethics Committee (AEC/04/2005/R370).

### Whole mount in situ hybridization and immunohistological analysis

Whole mount in situ hybridizations were performed essentially as described [65]. Pigmentation in embryos older than 24 hpf was inhibited either using PTU (I-phenyl-2-thiourea; Sigma) as described [63] or by bleaching in a 0.5 × SSC, 5% formamide, 10% H_2_O_2 _solution. For experiments where EGFP was detected by immunohistochemistry following whole mount in situ hybridization, the following modifications were used. Following Fast Red (Roche) staining, larvae were washed several times in PBS then transferred to blocking solution before binding of a rabbit anti-GFP-Alexa488 conjugated antibody (Molecular Probes) diluted 1:200 in blocking solution for 4 hours at room temperature. Larvae were then washed several times in PBS, fixed in 4% (w/v) PFA in PBS, mounted in 1% (w/v) low melting point agarose (Sigma) and images captured using a TCS SP2 confocal laser scanning microscope (Leica) through FITC (for Alexa488) and TRITC (for Fast Red stain) channels.

### Cloning of zebrafish *lysC *promoter and construction of pT2K/lysC::EGFP/DsRED2 constructs

A *lysC*-containing BAC clone (zC250A24) obtained from the RZPD (German Resource Center for Genome Research) was digested with *Xho*I and *Eco*RI which cut approximately 11,040 bp and 330 bp from the *lysC *initiation codon, respectively. This generated a 10.7 kb genomic fragment encompassing regulatory elements of the *lysC *gene starting 330 bp from the initiation codon. The remaining 3' sequence was generated by amplification using the following primer set: LysPA, 5'-CACCGGTGTCACTAATAACACGATCG-3' (which corresponded to sequence starting 30 nucleotides upstream of the *Eco*RI site); LysPB, 5'-TATACCCGGGAGGTGTATGGTGGAG-3' (which corresponded to sequence starting 39 nucleotides upstream of the initiation codon and contained a *Sma*I restriction site). This 330 bp PCR fragment was then cloned into the pGEM-T Easy Vector (Promega), sequence verified then digested with *Eco*RI and *Sma*I. This restriction fragment was cloned, together with the 10.7 kb *Xho*I/*Eco*RI fragment, into *Xho*I/*Sma*I-linearized pBS/KS to generate 11kblysCP/pBSKS. The 11 kb *lysC *promoter fragment was then liberated from 11kblysCP/pBSKS using *Xho*I and *Sma*I and ligated into *Xho*I/*Bam*HI(blunted)-linearized pT2KXIGΔin to generate pT2K/lysC::EGFP. The pT2K/lysC::DsRED2 construct was generated by first cloning the *Xho*I/*Sma*I-linearized 11 kb *lysC *promoter fragment from 11kblysCP/pBSKS together with a *Bam*HI (blunted)/*Kpn*I DsRED2-encoding construct into *Xho*I/*Kpn*I-linearized pBS/KS to create 11kblysCP/DsRED2/pBSKS. This construct was then digested with *Sac*II and *Xho*I to release 6.35 kb of *lysC *promoter sequence upstream of DsRED2 which was cloned into *Sac*II/*Xho*I-linearized pCS2+. Subsequent digestion with *Apa*I/*Cla*I facilitated the cloning of this 6.35 kb *lysC *promoter/DsRED2-encoding fragment into similarly linearized pT2KXIGΔin.

To confirm the efficiency with which the cloned lysC::EGFP/DsRED2 constructs could drive EGFP/DsRED2 expression and recapitulate the endogenous expression pattern of *lysC*, the transient expression profile of injected embryos was examined under fluorescence microscopy.

### Generation of transgenic *lysC::EGFP/DsRED2 *lines

Capped *transposase *transcript was generated using the mMESSAGE mMACHINE SP6 kit (Ambion Inc.) from the pCS-TP expression vector as previously described [66]. Zebrafish embryos were microinjected with the p2TK/lysC::EGFP/DsRED2 (30 pg/embryo) construct combined with *transposase *transcript (50 pg/embryo) at the 1-cell stage of development. These potential founders were then screened for reporter expression under fluorescence microscopy following 2 days of development. Only fluorescent embryos were selected to form the founder population. Once at sexual maturity, founders were intercrossed and F1 progeny screened for reporter expression. Pairs that generated a positive clutch were then individually outcrossed to wild type animals to identify the germline transgenic founder.

### Fluorescence microscopy

Images of whole transgenic embryos/larvae were generated using a DC200 digital camera and supporting software (Leica) connected to an MZFLIII fluorescence stereomicroscope equipped with GFP and DsRED filter sets (Leica).

### Microangiography

Microangiography was performed as described [67]. In brief, red fluorescent microspheres of 0.02 μm in diameter (Molecular Probes) were diluted 1:1 with a 2% BSA (Sigma) solution then sonicated. *LysC::EGFP *larvae to be injected were anaesthetized in tricaine as described [63] before being mounted in 1% (w/v) low melting point agarose (Sigma) in E3 medium ventral side up. The microsphere suspension was then injected either into the sinus venosus (for 2 dpf larvae) or directly into the heart (for 6 dpf larvae) using a FemtoJet pressure injection system (Eppendorf). The success of injection was monitored under an MZFLIII stereo microscope equipped with a DsRED filter set (Leica).

### FACS analysis, sorting and cytospin preparations

Cell collection, FACS analysis and sorting was performed essentially as described [44]. In brief, *lysC::EGFP *adult fish were anaesthetized in tricaine before dissection of kidneys which were dissociated by teasing through a 40 μm cell strainer (BD Falcon) in ice-cold 0.9 × PBS supplemented with 5% FBS. Cells were washed several times and resuspended in 0.9 × PBS/5% FBS before addition of propidium iodide to a final concentration of 1 μg/ml for exclusion of dead cells. FACS analysis was based on forward and side scatter characteristics, propidium iodide exclusion and GFP fluorescence using a FACS Vantage flow cytometer (Beckton Dickenson). Cytospin preparations were made using a Cytofuge 2 cytocentrifuge (StatSpin) followed by Leishman's staining for morphological analysis.

### Histology

Adult animals were anaesthetized in 4% paraformaldehyde, decalcified in 0.5 M EDTA (pH 7.8) for several days, dehydrated through an ethanol series, cleared in xylol overnight before infiltration and embedding in paraffin. Five-micrometer tissue sections were deparaffinized, rehydrated and immunohistochemical detection of EGFP performed as previously described [63] using a rabbit anti-GFP (Torrey Pines Biolabs Inc.) as a primary antibody and a HRP-conjugated anti-rabbit antibody (Sigma) as a secondary. Sections were counter stained with eosin.

### Injection of morpholino oligonuleotides

Morpholino oligonucleotides (Gene Tools, Philomath, OR) used in this study and their sequence are as follows: *gata1*ATGMO, 5'-CTGCAAGTGTAGTATTGAAGATGTC-3' [48] and *scl*spliceMO, 5'-AATGCTCTTACCATCGTTGATTTCA-3' [68]. MOs were injected essentially as described [69]. Effective doses for each morpholino were as described [48,68,69].

### Inflammation assay

The inflammation assay was conducted essentially as described [19]. In brief, 7 dpf *lysC::EGFP *larvae were anaesthetized in E3 medium supplemented with tricaine before the posterior portion of the developing caudal fin was transected with a sterile scalpel. Larvae were then left to recover in E3 medium before analysis under fluorescence microscopy using an MZFLIII stereo microscope equipped with a GFP filter set (Leica) during the progression of inflammation. The high resolution inflammation assay was performed by making a small incision in the ventral fin of anaesthetized 6 dpf *lysC::DsRED2*/*fli1::EGFP *compound transgenic larvae using a sterile scalpel followed by mounting in 1% low melting point agarose (Sigma) in E3 medium and time-lapse confocal microscopy using an Olympus FV1000 confocal microscope equipped with a heated chamber which was kept at 29°C and water-immersion lenses. Z-series were collected at 1 minute intervals and were no greater than 4 μm between each section as to ensure detection of all DsRED2-labeled cells. Projections of summed Z stacks, time-lapse animations and cell tracking were generated using ImageJ [70].

### Phagocytosis assay

Red fluorescent microspheres of 2 μm diameter (Molecular Probes) were selected to monitor the phagocytic ability of EGFP-expressing cells within 4 dpf *lysC::EGFP *larvae. Larvae to be injected were anaesthetized in tricaine then mounted in 1% (w/v) low melting point agarose (Sigma) in E3 medium. The microspheres were diluted 3:1 in sterile distilled water then microinjected into the embryonic tissue of 4 dpf transgenic larvae using a FemtoJet pressure injection system (Eppendorf). Injections were typically 0.5 μl in volume and targeted just posterior to the swim bladder within the somitic tissues. The success of injections was confirmed through fluorescence microscopy using an MZFLIII stereo fluorescence microscope equipped with a DsRED filter set (Leica). Phagocytosis of the individual microspheres was detected using a TCS SP2 confocal laser-scanning microscope (Leica) using GFP and TRITC channels. Projections of summed Z stacks and time-lapse animations were generated using ImageJ [70].

### Bacterial infection assay

GFP-labeled *Salmonella enterica *serovar Typhimurium were used to infect zebrafish. To label *Salmonella*, the strain SM022 [71] was recreated by P22 transduction [72] of the *rpsM*::*gfp *fusion and linked kanamycin resistance genes from SM022 into the original parental *Salmonella *strain SL1344 [71]. Five dpf *lysC::DsRED2 *larvae (raised in E3 medium supplemented with PTU to inhibit pigmentation, as described [63]) were immersed in 2.93 × 10^9^/ml CFU GFP-labeled *Salmonella *strain SL1344. Following a 24 hour infection period surviving larvae were washed several times in PTU-supplemented E3. Uptake of GFP-expressing *Salmonella *was monitored through fluorescence microscopy. Successfully infected transgenic larvae were then anaesthetized, mounted in 1% (w/v) low melting point agarose (Sigma) and imaged using an Olympus FV1000 confocal microscope equipped with a heated chamber which was kept at 29°C.

## Authors' contributions

CH carried out experimental work and drafted the manuscript. TS performed bacterial infection assay. MVF, PC and KC all provided significant contributions to the conception and design of the study as well as critically reviewing the manuscript. All authors read and approved the final manuscript.

## Supplementary Material

Additional file 1 ***lysC *expression during early embryonic/larval development**. *lysC *expression within 22 hpf (A and B), 30 hpf (C-E), 36 hpf (F-H), 48 hpf (I-K), 4 dpf (L-N), 5 dpf (O and P) and 7 dpf (Q and R) zebrafish embryos and larvae. Arrow in D denotes *lysC*-expressing cell within head mesenchyme. Arrows in O-Q denote *lysC *transcripts within the developing pronephric glomerulus. Scale bars: 200 μm. Click here for file

Additional file 2 **Co-localized expression of EGFP and DsRED2 within *lysC::EGFP*/*lysC::DsRED2 *compound transgenic larvae**. (A) Summed Z-stacks through ICM compartment of EGFP and DsRED2 expression (and merged images) within 48 hpf *lysC::EGFP*/*lysC::DsRED2 *larva. (B) Higher magnification within same region. Scale bars: 50 μm in A; 10 μm in B. Click here for file

Additional file 3 **Movie of dividing EGFP-labeled cell within ICM compartment of 52 hpf *lysC::EGFP *larva**. Each frame represents summed Z-stacks through a single EGFP-labeled cell collected every 30 seconds over a 37 minute period. The dividing cell is initially marked by a blue dot. Click here for file

Additional file 4 ** Movie of labeled cell rolling along vascular endothelia**. Each frame represents summed Z-stacks through a single DsRED2-expressing cell collected every 20 seconds (over an approximate 9 minute period) as it rolls along the luminal endothelium of the caudal vein within a 6 dpf *lysC::DsRED2*/*fli1::EGFP *compound transgenic larva, anterior to left. For reference the movement is tracked with a blue line. Click here for file

Additional file 5 **Labeled cells are located within the developing brain and retina**. (A-C and D/E) Lateral and dorsal bright field, GFP and merged views (of summed Z-stacks), respectively, of EGFP-labeled cells within the developing hindbrain (A and D), midbrain (B), retina (C) and forebrain (E) within 36 hpf (A and B), 48 hpf (C) and 2.5 dpf (D and E) *lysC::EGFP *animals. Anterior to left in all images. Abbreviations: E, eye; Le, lens; Ol, olfactory organ; OV, otic vesicle; Re, retina. Scale bars: 100 μm in A/B/D and E; 25 μm in C. Click here for file

Additional file 6 **Labeled cells are restricted to the myeloid lineage**. (A and B) EGFP expression within 48 hpf *lysC::EGFP *transgenic larvae. (C and D) EGFP expression within 48 hpf *lysC::EGFP *transgenic larvae following early delivery of *scl*-targeting MOs. Arrows in D denote pericardial edema. (E and F) EGFP expression within 28 hpf *lysC::EGFP *embryo and 28 hpf transgenic embryo following early delivery of *gata1*-targeting MOs, respectively. (A/C/E/F and B/D) Lateral views of tail/trunk and dorsal views of cranio-trunk region, respectively, anterior to left. Scale bars: 200 μm. Click here for file

Additional file 7 **Movie of tracked cell demonstrating retrograde chemotaxis**. Each frame represents Z-stacks collected every minute during an inflammatory response within 6 dpf *lysC::DsRED2*/*fli1::EGFP *compound transgenic animals following wounding of the ventral fin. The migratory path of the DsRED2-labeled cell is highlighted by a blue line as it exits from the EGFP-labeled vasculature towards the wound and then back into the circulation. White polygon demarcates injury boundaries. Movie played at 7 frames/second. Click here for file

Additional file 8 **Movie of tracked cell that infiltrates the injury site and returns to the vasculature twice**. Each frame represents Z-stacks collected every minute during an inflammatory response within 6 dpf *lysC::DsRED2*/*fli1::EGFP *compound transgenic animals following wounding of the ventral fin. The migratory path of the DsRED2-labeled cell is highlighted by a blue line as it returns to the circulation then re-visits the wound followed by migration back towards the vasculature. White polygon demarcates injury boundaries. Movie played at 7 frames/second. Click here for file

Additional file 9 **Movie of tracked cell that fails to infiltrate the wound**. Each frame represents Z-stacks collected every minute during an inflammatory response within 6 dpf *lysC::DsRED2*/*fli1::EGFP *compound transgenic animals following wounding of the ventral fin. The migratory path of the DsRED2-labeled cell is highlighted by a blue line as it initially emerges from the caudal vein and migrates towards the injury only to cease this trajectory and return to the vasculature. White polygon demarcates injury boundaries. Movie played at 7 frames/second. Click here for file

Additional file 10 **Movie of interacting EGFP-labeled cells within 4 dpf *lysC::EGFP *larva**. Time-lapse confocal microscopy of interacting EGFP-labeled cells within 4 dpf *lysC::EGFP *larva. Movie of two EGFP-expressing cells (one containing a phagocytosed microsphere) over an approximate 7.5 minute interval (imaged every 20 seconds). Movie played at 5 frames/second. Click here for file

Additional file 11 **Infection with GFP-expressing *Salmonella *results in a robust inflammatory response within the posterior intestine**. Summed Z-stacks through the posterior intestine of 8 dpf *lysC::DsRED2 *larva following infection at 5 dpf with GFP-expressing *Salmonella*. Arrows denote autofluorescence of pigment cells. Scale bar: 50 μm. Click here for file
