# Supplementary figures and images for: The zebrafish lysozyme C promoter drives myeloid-specific expression in transgenic fish
Source: BMC Dev Biol. 2007 May 4;7:42. doi: 10.1186/1471-213X-7-42 (PMC1877083; doi:10.1186/1471-213X-7-42)

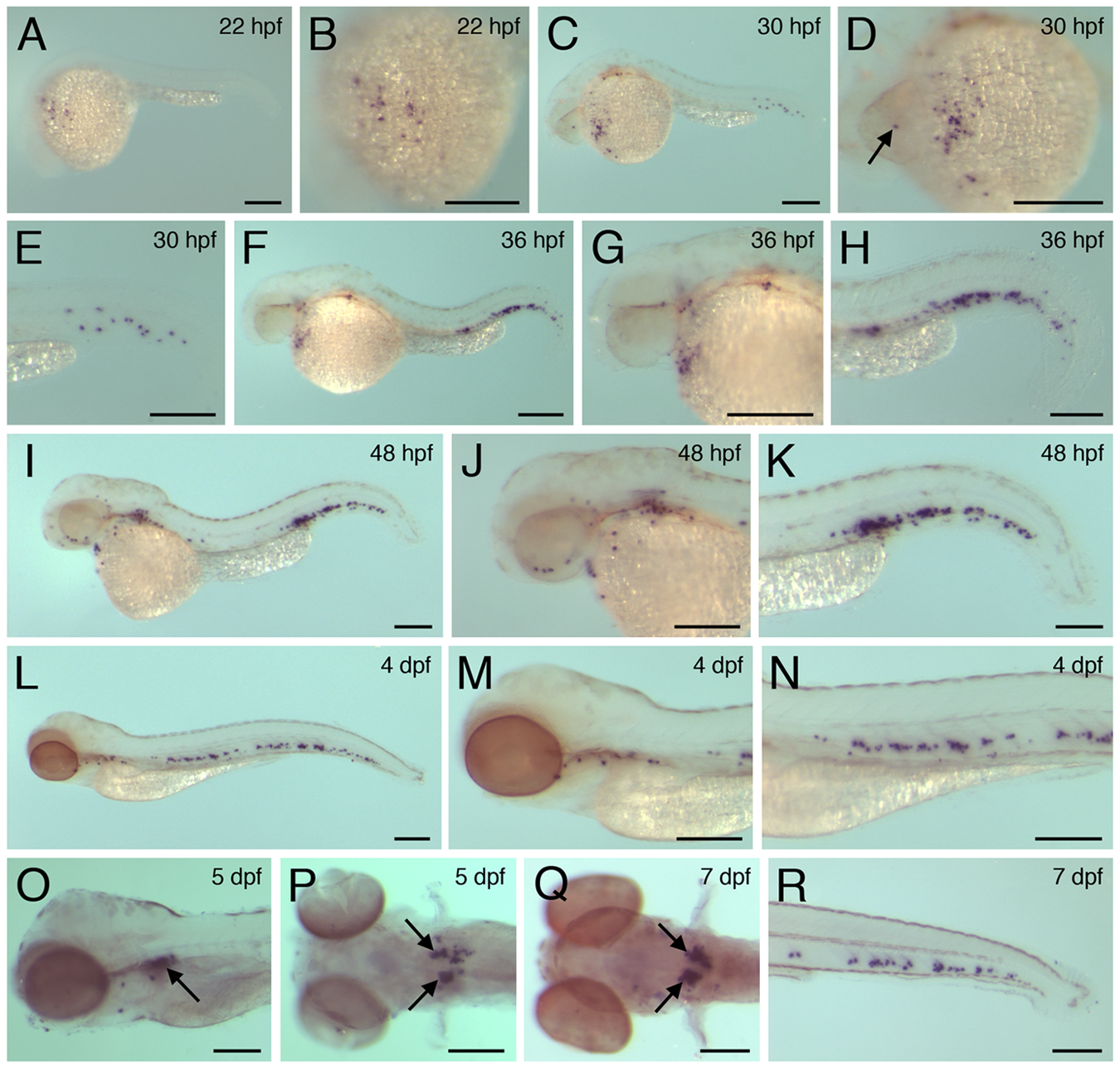

Supplement: Additional file 1 — lysC expression during early embryonic/larval development. lysC expression within 22 hpf (A and B), 30 hpf (C-E), 36 hpf (F-H), 48 hpf (I-K), 4 dpf (L-N), 5 dpf (O and P) and 7 dpf (Q and R) zebrafish embryos and larvae. Arrow in D denotes lysC-expressing cell within head mesenchyme. Arrows in O-Q denote lysC transcripts within the developing pronephric glomerulus. Scale bars: 200 μm. [file 1471-213X-7-42-S1.tiff]

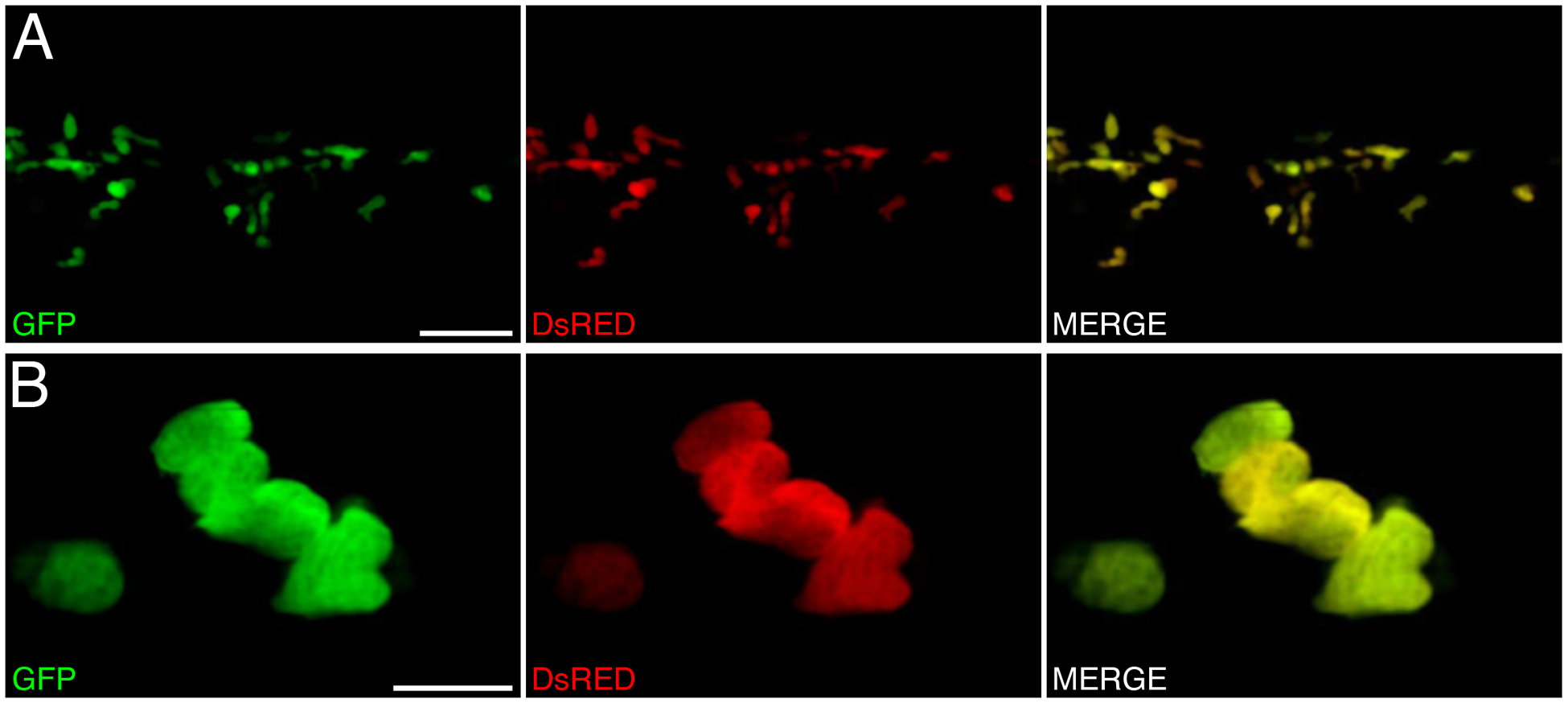

Supplement: Additional file 2 — Co-localized expression of EGFP and DsRED2 within lysC::EGFP/lysC::DsRED2 compound transgenic larvae. (A) Summed Z-stacks through ICM compartment of EGFP and DsRED2 expression (and merged images) within 48 hpf lysC::EGFP/lysC::DsRED2 larva. (B) Higher magnification within same region. Scale bars: 50 μm in A; 10 μm in B. [file 1471-213X-7-42-S2.tiff]

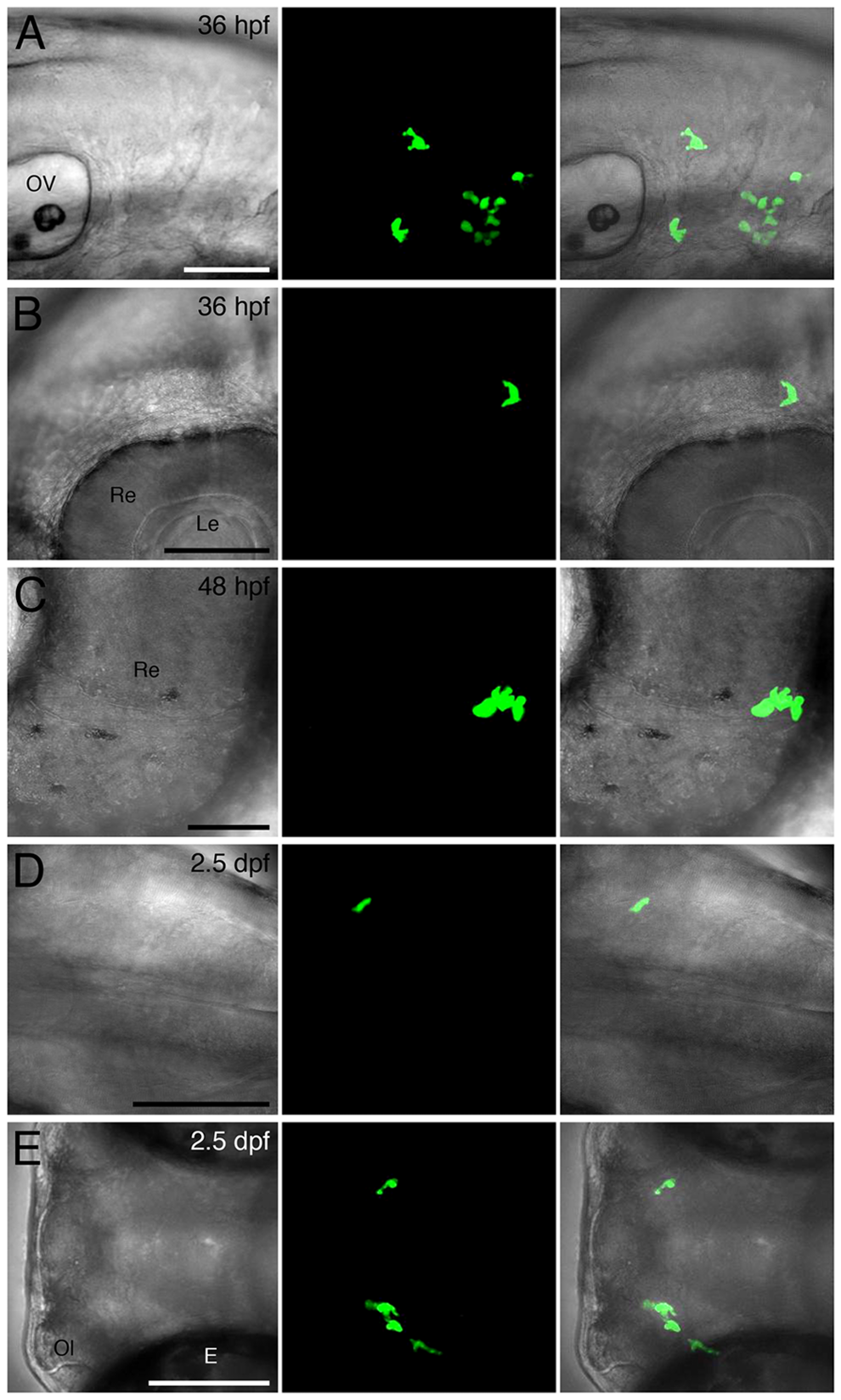

Supplement: Additional file 5 — Labeled cells are located within the developing brain and retina. (A-C and D/E) Lateral and dorsal bright field, GFP and merged views (of summed Z-stacks), respectively, of EGFP-labeled cells within the developing hindbrain (A and D), midbrain (B), retina (C) and forebrain (E) within 36 hpf (A and B), 48 hpf (C) and 2.5 dpf (D and E) lysC::EGFP animals. Anterior to left in all images. Abbreviations: E, eye; Le, lens; Ol, olfactory organ; OV, otic vesicle; Re, retina. Scale bars: 100 μm in A/B/D and E; 25 μm in C. [file 1471-213X-7-42-S5.tiff]

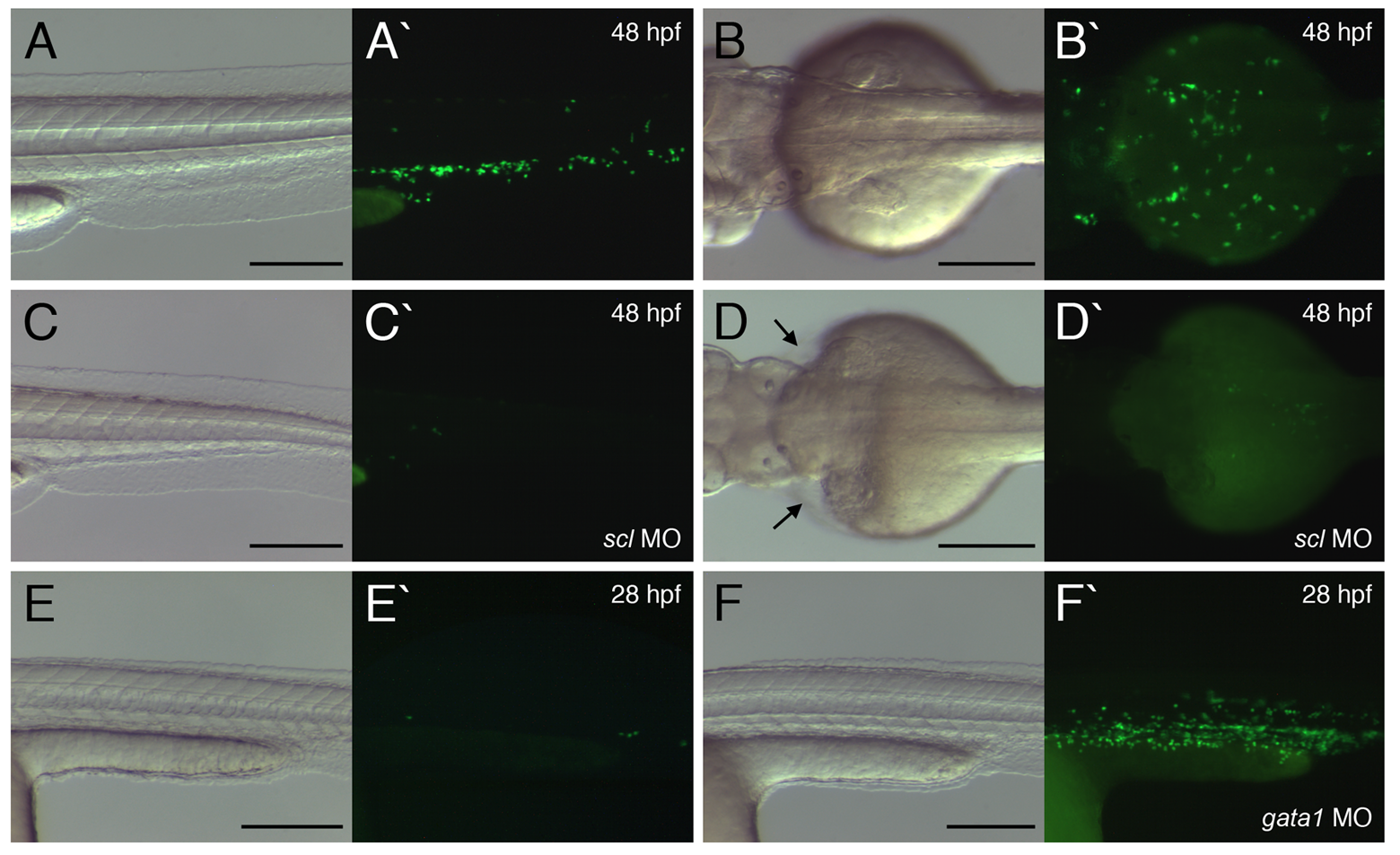

Supplement: Additional file 6 — Labeled cells are restricted to the myeloid lineage. (A and B) EGFP expression within 48 hpf lysC::EGFP transgenic larvae. (C and D) EGFP expression within 48 hpf lysC::EGFP transgenic larvae following early delivery of scl-targeting MOs. Arrows in D denote pericardial edema. (E and F) EGFP expression within 28 hpf lysC::EGFP embryo and 28 hpf transgenic embryo following early delivery of gata1-targeting MOs, respectively. (A/C/E/F and B/D) Lateral views of tail/trunk and dorsal views of cranio-trunk region, respectively, anterior to left. Scale bars: 200 μm. [file 1471-213X-7-42-S6.tiff]

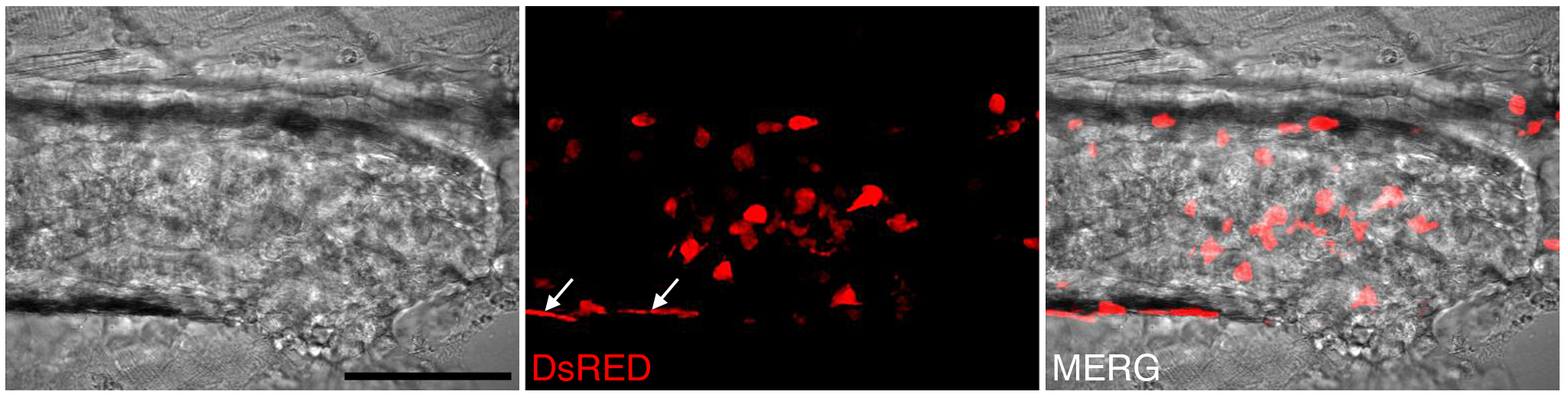

Supplement: Additional file 11 — Infection with GFP-expressing Salmonella results in a robust inflammatory response within the posterior intestine. Summed Z-stacks through the posterior intestine of 8 dpf lysC::DsRED2 larva following infection at 5 dpf with GFP-expressing Salmonella. Arrows denote autofluorescence of pigment cells. Scale bar: 50 μm. [file 1471-213X-7-42-S11.tiff]
